# Chronic Heat Stress Induces Oxidative Stress and Induces Inflammatory Injury in Broiler Spleen via TLRs/MyD88/NF-κB Signaling Pathway in Broilers

**DOI:** 10.3390/vetsci11070293

**Published:** 2024-07-01

**Authors:** Haoxiang Chen, Feiyao Wang, Xingyue Wu, Songchen Yuan, Huili Dong, Chenyang Zhou, Siliang Feng, Zhanqin Zhao, Lifang Si

**Affiliations:** College of Animal Science and Technology, Henan University of Science and Technology, Luoyang 471000, China

**Keywords:** heat stress (HS), broiler, spleen, antioxidant, TLRs, NF-κB

## Abstract

**Simple Summary:**

Heat stress (HS) is currently one of the critical problems facing the global farmed broiler farming industry. After chronic HS, broiler production performance decreases, the body’s antioxidant capacity decreases, oxidative stress occurs, and damage occurs in the splenic tissues, which causes an inflammatory response in the spleen through the inflammatory pathway, leading to a decline in immune function, which seriously affects the health of broilers. This is important for exploring the drugs that inhibit inflammatory responses to mitigate the harm HS triggers. This is important for exploring drugs to inhibit the inflammatory response to alleviate the damage caused by HS and to reduce the loss caused by HS in broiler breeding.

**Abstract:**

The spleen is the largest peripheral immune organ of the organism, accounting for 25% of the total lymphoid tissue of the body. During HS, the spleen is damaged due to the elevated environment, which seriously affects life performance and broilers’ health. This study aimed to investigate the mechanism of chronic HS damage to broiler spleen tissues. The broilers were typically raised until they reached 21 days of age, after which they were arbitrarily allocated into two groups: an HS group and a cntrol group. The HS group was subjected to a temperature of 35 °C for 10 h each day, starting at 21 days of age. At 35 and 42 days of age, spleen and serum samples were obtained from the broilers. The results showed that after HS, a significant decrease in productive performance was observed at 42 days of age (*p* < 0.01), and the spleen index, and bursa index were significantly decreased (*p* < 0.01). T-AOC of the organism was significantly decreased (*p* < 0.05), GSH-PX, SOD, and CAT antioxidant factors were significantly decreased (*p* < 0.01), and MDA was significantly elevated (*p* < 0.01). HS also led to a significant increase in cytokines IL-6, TNF-α, and INF-γ and a significant decrease in IL-4 in the spleen. The histopathologic results showed that the spleen’s red-white medulla was poorly demarcated. The cells were sparsely arranged after HS. After HS, the expression of TLRs, MYD88, and NF-κB genes increased significantly. The expression of HSP70 increased significantly, suggesting that HS may induces an inflammatory response in broiler spleens through this signaling pathway, which may cause pathological damage to broiler spleens, leading to a decrease in immune function and progressively aggravating HS-induced damage with the prolongation of HS.

## 1. Introduction

Heat Stress (HS) has become one of the biggest challenges, jeopardizing the global broiler farming industry. The probability of HS is also higher for poultry farms due to the higher density of poultry farming and more feathered poultry. HS can cause serious harm to the growth and development of poultry, resulting in growth retardation and decreased production performance [1]. Especially in hot summers, the body temperature of poultry rises significantly, inhibiting the intestinal tract’s digestive function and maybe even leading to immunosuppressive diseases [2]. Heat Shock Protein 70 (HSP70) and Heat Shock Protein 90 (HSP90) are significantly elevated in animals that receive thermal stimulation, which results in sympathetic arousal and increased secretion of adrenal medulla and corticotropin [3]. Peripheral blood adrenal glucocorticoids are secreted by the hypothalamic–pituitary–adrenocortical axis in animals. The glucocorticoids are mainly cortisol and corticosterone (CORT) [4]. Whereas the main glucocorticoid in poultry is CORT, long-term chronic HS elevates CORT in poultry blood [5]. That is why blood HSP70, HSP90, and CORT are often used as indicators of HS in poultry.

Oxidative Stress (OS) refers to the increased release of Reactive Oxygen Species (ROS) in animal organisms under different stimulatory factors. This damages protein, lipids, and DNA, induces lipid peroxidation and protein denaturation, further enhances ROS production, inhibits the activity of antioxidant enzymes, and disrupts intracellular homeostasis [6]. HS is a major environmental factor leading to oxidative stress and associated tissue damage [7,8]. It can disrupt mitochondrial homeostasis, induce the excessive release of ROS and cellular dysfunction, and cause apoptosis, leading to tissue and organ damage [9].

The inflammatory response is the body’s defense against external stimuli or stimuli within the body. It has an essential regulatory role in maintaining the normal physiological functions of the body and cells, in which several signaling pathways are involved in the inflammatory response. However, the main ones in poultry are the NF-κB and TLR/MYD88 signaling pathways [10].

TLR/MyD88 is one of the essential signaling pathways of the immune system, and plays a vital role in activating the body’s innate immunity and inducing the defense mechanism of adaptive immunity [11]. Toll-like Receptors (TLRs) and Myeloid Differentiation Factor 88 (MyD88) are upstream regulators of Nuclear Factor-κB (NF-κB). TLRs are a pattern recognition family involved in lipopolysaccharide (LPS) recognition and signaling that elicit pathological responses in response to LPS [12]. It has been shown that when TLRs receive stimulus signals, they trigger intracellular pathways that produce cytokines and chemokines that defend against the stimulation of stressors [13]. TLR4, the first TLR protein identified, is an essential member of the TLR family and plays a vital role in various inflammatory responses. It has been found that TLRs can bind to LPS, triggering an inflammatory signaling cascade response that ultimately leads to the production of pro-inflammatory cytokines by the body [14].

MyD88 is a crucial connector molecule in the signaling pathway of TLRs and is an essential signaling gene linking innate and adaptive immune responses [15,16]. The structure of MyD88 consists of three functional regions: (i) the C-terminal TIR structural domain, (ii) the intermediate domain (INT), and (iii) the N-terminal death domain (DD) [17]. The C-terminal TIR structural domain is mainly responsible for signaling downstream by binding to proteins. The DD region mainly recruits downstream signaling molecules with death structural domains into downstream signaling. It has been found that only the simultaneous expression of the DD region and the INT region in MyD88 can activate the downstream NF-κB pathway, while other combinations cannot [18,19]. MyD88 acts as a junction protein involved in immune responses mediated by most members of the TLRs family [20]. It was found that in a wild-type mouse model of colitis-associated cancer, blockade of the TLR4/MyD88 signaling pathway using a MyD88 inhibitor reduces the proliferative capacity and promotes apoptosis of colonic epithelial cells, decreases serum TNF-α and IL-6 secretion, alleviates the inflammatory response of the intestinal tract, and inhibits tumor formation [21]. The above studies have shown that MyD88 is involved in various inflammatory diseases in the organism and participates in various activities, such as cytokine secretion and proliferation and apoptosis of inflammatory cells in vivo. However, relatively few studies have been conducted on the inflammatory response generated by HS, so it is of great significance to explore the damage caused by HS to the broiler organism through activation of the TLRs/MyD88/NF-κB signaling pathway [22].

The NF-κB pathway is a crucial pro-inflammatory signaling mechanism. NF-κB can selectively bind to the regulatory regions of the genes encoding the κ-light chain of B cells, thus playing a role in the immune response of the cells [23]. The primary process of NF-κB transduction is as follows: when internal or external stimuli do not stimulate the cell, the NF-κB dimer forms a relatively stable complex with IκB in the cytoplasm [24]. When the cell is stimulated, the NF-κB dimer dissociates from IκB proteins and is rapidly activated. The activated NF-κB transmits the signals to the nucleus and serves as a transcription factor to enhance the transcription of TNF-α and IL-1β genes [25], followed by an increase in the secretion and release of pro-inflammatory cytokines IL-6 and IFN-α, which leads to further amplification of the inflammatory response [26,27].

The spleen is one of the peripheral immune organs of the organism, is the largest lymphatic organ of the organism, with immunologically active T-cells and B-cells, and is also the place of reaction to receive antigenic stimulation to produce an immune response [28]. The development and function of the spleen determine the level of immune function in broilers. HS can cause structural damage and dysfunction of the broiler spleen, resulting in an inflammatory response of the spleen. However, the mechanism of action could be more precise, and how TLRs are involved in inflammatory injury of the spleen in heat-stressed broilers is still being determined. Thus, in this study, the HS broiler model was replicated. To detect the production performance, histopathological sections were used to observe the pathological changes in the spleen, Enzyme-linked Immunosorbent Assay (ELISA) was used to detect the expression of CAT, SOD, GSH-Px, T-AOC, and MDA, and real-time fluorescence quantitative PCR was used to detect the expression levels of TLR2, TLR4, MyD88, TRIF, and F-κB, and the expression levels of the cytokines IL-4, IL-6, TNF-α, and IFN-γ mRNA.

## 2. Materials and Methods

### 2.1. Animals and Treatment Design

The experiment utilized a parallel group design. One-hundred-and-twenty 1-day-old AA male broilers were purchased from broiler hatcheries and raised to 21 days of age according to the appropriate temperature and humidity for broilers, and standard inoculation procedures were used. At 21 days of age, 100 broilers were randomly selected and divided into control and Heat Stress (HS) groups, with five replicates of 10 chickens each. The temperature was maintained at 23 ± 1 °C in the control group and 35 ± 1 °C during HS in the HS group, which started at 9 a.m. and continued until 7 p.m., with HS lasting for 10 h per day, and the temperature was maintained at 23 ± 1 °C for the rest of the day. Humidity was maintained at 55 ± 5% in both HS and cntrol groups. All broilers were given standard feed and water, and the composition of the elemental diets is listed in Table 1. All broilers were raised in cages (length × width × height, 200 × 75 × 70 cm) that could accommodate 10 broilers, all of which were allowed to eat and drink freely. Regular cleaning and disinfection of chicken coops were performed.

### 2.2. Sample Collection

Serum and spleen tissues were collected at 35 and 42 days of age, respectively. At 35 and 42 days of age, one chicken from each replicate was randomly selected for wing vein blood collection, followed by intravenous injection of 5% pentobarbital sodium, euthanasia of broiler chickens, and then collection of spleen tissues. The collected blood was centrifuged to collect serum and stored at −25 °C. The spleen tissue was divided into three parts: one was placed in 4% paraformaldehyde, one in an electron microscope fixative, and the other was transferred to −80 °C for storage.

### 2.3. Determination of Productive Performance and Organ Index

During the experimental period, broilers were weighed on an empty stomach at 8:00 a.m. on days 21 and 42 after 12 h of fasting, and each group’s average daily feed intake was recorded. The final body weights of broilers during the experimental period were measured during sampling. Then, the immune organs were weighed on 0.001 g electronic scales. The production performances and immune organ indices were calculated. Production performance and immune organ index were calculated as follows:

Average daily feed intake (ADFI) = feed consumption/(age of surviving chickens + age of dead chickens)

Average daily weight gain (ADG) = (total weight gain of surviving chickens + weight of dead chickens)/(age of surviving chickens + age of dead chickens)

Feed to weight ratio (F:G) = average daily feed intake/average daily weight gain

Immune organ index = Organ weight (g)/Live weight (kg) × 100%.

### 2.4. Pathological Analysis

Transmission electron microscopy: Broiler spleen tissues were taken and fixed in an electron microscope fixative, then post-fixed with 1% osmium acid, osmotically embedded by alcohol dehydration, and prepared as 80 nm ultramicrotomes. The samples were treated with uranyl acetate and lead citrate to create stains. Subsequently, the images were taken and examined using a transmission electron microscope.

HE staining: After fixation in 4% paraformaldehyde for 24 h, paraffin embedding was performed by dehydration, followed by preparing 5 μm paraffin sections. After the paraffin sections were dewaxed and dehydrated, the nuclei were stained blue with a hematoxylin staining solution. Then, the sections were put into 1% hydrochloric acid alcohol differentiation solution to differentiate and reblue solution to return the blue color. Finally, the sections were put into an eosin staining solution to stain the cytoplasm of the cells red. The sections were dehydrated by alcohol, and then the sections were sealed with neutral resin after being transparent by xylene. The spleen tissues were studied according to their morphological features using a light microscope constructed by Olympus Corporation in Tokyo, Japa, to observe the morphological characteristics of the spleen tissue.

### 2.5. Serum Antioxidant Capacity and Corticosterone Detection

The enzyme activities of superoxide dismutase (SOD), glutathione peroxidase (GSH-Px), and catalase (CAT), as well as the total antioxidant capacity (T-AOC) and malondialdehyde (MDA) levels in serum, were measured using a specific kit provided by Nanjing Jiancheng Bioengineering Institute in Nanjing, China. The instructions provided with the kit were followed precisely. We used a CORT ELISA kit provided by Shanghai Enzyme Linked Biotechnology Co., Ltd. (Shanghai, China), and strictly followed the instructions.

### 2.6. Quantitative Real-Time Polymerase Chain Reaction Assay

The expression levels of TLR2, TLR4, MyD88, TRIF, IL-4, IL-6, TNF-α, INF-γ, HSP70, and HSP90 were detected in spleen tissues by qPCR. The RNA was isolated using a TRIzol reagent (Nanjing, China) following the directions provided by the manufacturer. The spectrophotometer was used to determine the concentration of total RNA and the A260/A280 ratio. The process of reverse transcription of total RNA into cDNA was carried out using a Hiscript III QRT Supermix kit (Nanjing, China) following the instructions provided by the manufacturer. A CFXX kit was utilized to conduct real-time quantitative polymerase chain reaction (PCR) experiments. Assays were performed using a CFX96 Touch System (Bio-Rad, Hercules, CA, USA). Quantitative PCR was performed with 5 μL of cDNA template, 0.4 μL of forward primer, 0.4 μL of reverse primer, 4.2 μL of dH_2_O, and 10 μL of SYBR qPCR Master mix, i.e., a total system of 20 μL. The internal control used was β-actin. The PCR protocol involved an initial denaturation step at a temperature of 95 °C for 30 s, followed by 40 cycles at 95 °C, an initial denaturation at 95 °C for 30 s, a denaturation at 95 °C for 10 s, an annealing at 60 °C for 30 s, an extension at 95 °C for 15 s, an extension at 60 °C for 60 s, and finally an extension at 95 °C for 15 s. All primers used in this study were used in a single reaction volume. The primers used for the present investigation were created employing the PrimerQuest program (https://eu.idtdna.com/pages, accessed on 15 October 2023.). The cDNA sequences were downloaded from NCBI’s nucleotide database (https://www.ncbi.nlm.nih.gov/nucleotide/, accessed on 15 October 2023.). Complete details concerning the primers are to be found in Table 2.

### 2.7. Statistical Analysis

The statistical analyses of a single-way ANOVA and *t*-tests for distinct samples were performed using IBM SPSS Statistics 26 software. The data were provided as the mean value ± the Standard Error (SE). A level of significance of *p* < 0.05 was deemed significant. In contrast, a level of significance of *p* < 0.01 was regarded as highly significant.

## 3. Results

### 3.1. Determination of Productive Performance and Organ Index

In order to investigate the effect of continued exposure to scorching heat on the performance of broiler chickens, we measured their body weight at the onset of HS at 21 days old and the conclusion of HS at 42 days old. The findings, as presented in Table 3, indicated that at 42 days of age, the HS group presented a remarkable drop in body weight, mean daily weight gain, and average daily feed consumption (*p* < 0.01), along with a significant increase in the feed-to-weight ratio (*p* < 0.01), in contrast with the control group.

To investigate the effect of chronic HS on immune organ indices in broilers, we determined the immune organ indices at 42 days of age, and the results, as shown in Table 4, showed that the thymus index was significantly lower in the HS group compared with the control group (*p* < 0.05), and the spleen index and bursa index were significantly lower (*p* < 0.01).

### 3.2. Histopathologic Analysis of the Spleen

**Figure 1:**
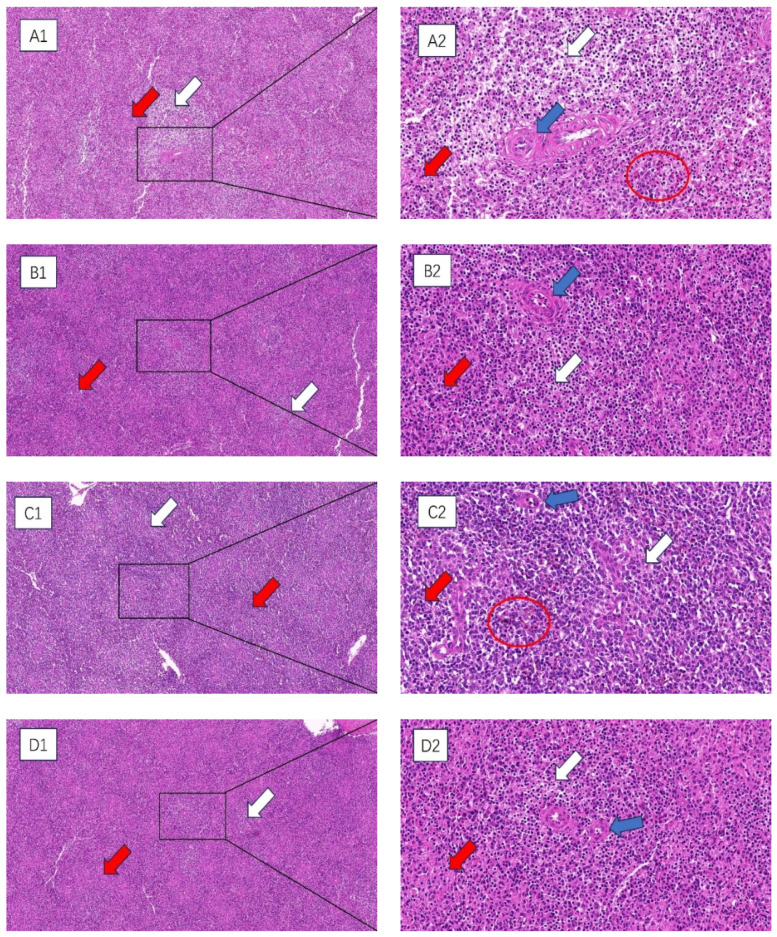
The impact of chronic HS results in histological changes in the spleen of broiler chickens. (**A1**–**D1**) 100× field of view (**A2**–**D2**) 400× field of view. White arrow: white medulla oblongata. Red arrow: red medulla oblongata. Blue arrow: central artery. Red circle: splenic hemorrhage. (**A1**,**A2**) 35-day-old HS group, (**B1**,**B2**) 35-day-old control group, (**C1**,**C2**) 42-day-old HS group, (**D1**,**D2**) 42-day-old control group.

HE staining was used to observe the histopathological changes in the spleens of broilers subjected to chronic HS, and the results are shown in Figure 1. At 35 and 42 days of age, compared with the control group, the spleens of broilers in the HS group were hemorrhagic. The tissues appeared to have atrophic changes, with a greater degree of morphologic changes in the histological structure, a more chaotic structural arrangement of the cells, more expansive interstitial spaces between the cells of the spleens, and a looser arrangement of the cells, and the appearance of precipitated aggregates of amyloid material. The boundary between the red and white medulla of the spleen was very blurred, and the splenic microsomes’ boundary was unclear.

**Figure 2:**
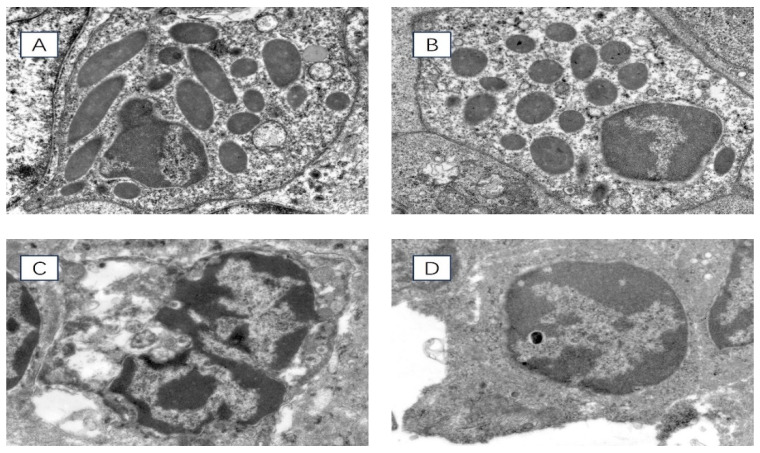
Ultrastructure of chicken spleen cells under transmission electron microscope 8000× field of view. (**A**) 35-day-old HS group, (**B**) 35-day-old control group, (**C**) 42-day-old HS group, (**D**) 42-day-old control group.

Transmission electron microscopy was used to observe the ultrastructure of spleen cells in chronically heat-stressed broilers, and the results are shown in Figure 2. At 35 days of age, the nuclear membranes of the spleen cells in the HS group were disrupted. The nuclear chromatin exhibited more significant amounts of condensation than the control group. At 42 days of age, the nuclear membranes of the spleen cells in the HS group experienced significant rupture. The nuclear chromatin appeared to be severely condensed compared with the control group.

### 3.3. Analysis of Serum Indicators

**Figure 3:**
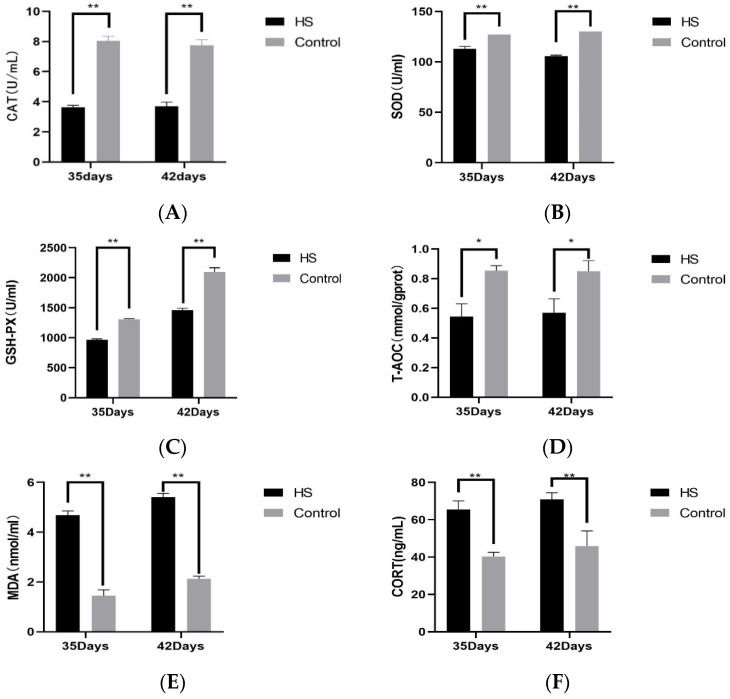
Effect of chronic HS on CAT (**A**), SOD (**B**), GSH-PX (**C**), T-AOC (**D**), MDA (**E**), and CORT (**F**) expression in broiler serum. HS: Heat stress group. Control: Blank control group. Data are expressed as mean ± SE (n = 5) (* *p* < 0.05, ** *p* < 0.01).

We examined the concentrations of CAT (A), SOD (B), GSH-PX (C), T-AOC (D), MDA (E), and CORT (F) in the serum of broiler chickens at 35 and 42 days of age. The results are shown in Figure 3. At 35 and 42 days of age, CAT, SOD, GSH-PX, and T-AOC in the HS group were significantly lower than those of the control group (*p* < 0.05). At 35 and 42 days of age, the concentrations of MDA and CORT were significantly higher in the HS group than in the control group (*p* < 0.01).

### 3.4. Expression of Related Genes in Spleen

**Figure 4:**
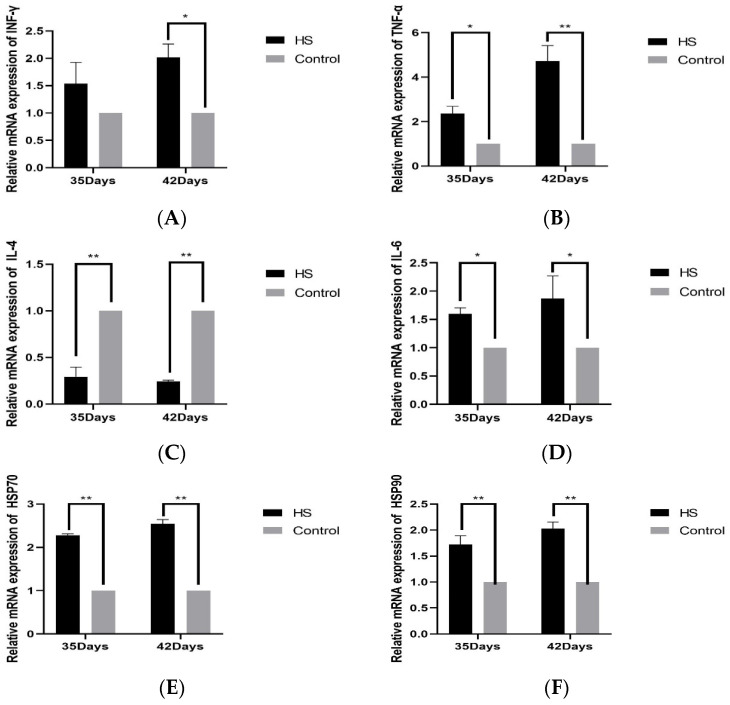
Effects of chronic HS on mRNA expression of INF-γ (**A**), TNF-α (**B**), IL-4 (**C**), IL-6 (**D**), HSP70 (**E**), and HSP90 (**F**) in broiler spleen. HS: HS group. Control: Blank control group. Data are expressed as mean ± SE (n = 5) (* *p* < 0.05, ** *p* < 0.01).

The relative expression of cytokines INF-γ (A), TNF-α (B), IL-4 (C), IL-6 (D), HSP70 (E), and HSP90 (F) in spleen tissues was detected by PCR. The results will be shown in Figure 4. The relative levels of INF-γ in the HS group at 35 days of age were not significantly varied above that of the control group (*p* > 0.05). The 42-day-old group was significantly higher than the control group (*p* < 0.05). The expression of TNF-α was considerably higher in the HS group compared to the control group at 35 and 42 days of age (*p* < 0.05). It was significantly higher than that of the control group at 42 days of age in HS groups (*p* < 0.01), the relative expression of IL-4 was significantly lower than that of the control group at 35 and 42 days of age in HS groups (*p* < 0.01), and the relative expression of IL-6 was significantly higher than that of the control group at 35 and 42 days of age in HS groups (*p* < 0.05). The relative expression of HSP70 and HSP90 was significantly higher in the HS group than in the control group at 35 and 42 days of age (*p* < 0.01).

**Figure 5:**
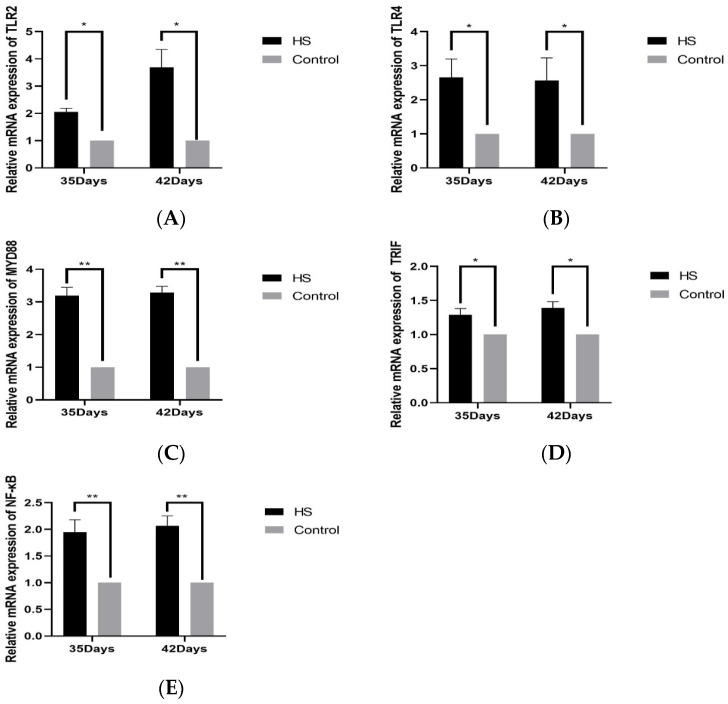
Effects of chronic HS on mRNA expression of TLR2 (**A**), TLR4 (**B**), MYD88 (**C**), TRIF (**D**), and NF-κB (**E**) in broiler spleen. HS: Heat stress group. Control: Blank control group. Data are expressed as mean ± SE (n = 5) (* *p* < 0.05, ** *p* < 0.01).

The relative expression of TLR2 (A), TLR4 (B), MYD88 (C), TRIF (D), NF-κB (E), and HSP70 (F) in spleen tissues was detected by PCR, as shown in Figure 5. The relative expression of TLR2 and TLR4 was significantly higher in the HS group than in the control group at 35 days of age and 42 days of age (*p* < 0.05); the relative expression of MYD88 and NF-κB was significantly higher in the HS group than the control group at 35 and 42 days of age (*p* < 0.01). The relative expressions of TRIF and HSP70 were significantly lower in the HS group than in the control group at 35 and 42 days of age (*p* < 0.05).

## 4. Discussion

Heat Stress (HS) is a prevalent issue worldwide in broiler farming. Research has demonstrated that HS can harm many organs, disrupting their regular operations [29]. However, less research has been conducted on the mechanism of HS injury to the broiler spleen, which is the largest peripheral immune organ of the organism [30], accounting for 25% of the total body lymphoid tissues and comprising a substantial quantity of lymphocytes and macrophages, which serve as the center of gravity for the cellular and humoral immune responses in the body, w ith immunologically active T and B cells and the response to receiving antigenic stimuli to generate an immune response site [28].

High temperature can activate the sympathetic adrenomedullary system, prompting hypothalamus, pituitary, and adrenal cortex hyperactivation, leading to increased secretion of adrenaline glucocorticoids and other hormones, which causes metabolic disorders in the organism. There was a decrease in performance, namely in terms of growth rate and feed conversion ratio [31]. It was found that when the ambient temperature exceeded 30 °C, for every 1 °C rise in temperature, broilers’ feed intake decreased by 4.6% [32]. HS accelerates glycolysis reaction, leading to protein and fat decomposition and shortening the retention time of surimi in the digestive tract, which affects daily weight gain and feed-to-weight ratio [33]. The immune organ index can reflect the immunity level of the broiler’s organism, an important indicator of immune organ development. A decrease in the absolute weight of organs and organ index both indicate that the immune function of the organism is affected to some extent. At the same time, chronic HS causes edema and hemorrhage of immune organs, and a significant decrease in immune organ weight and organ index in broiler chickens. It is closely related to the duration of HS [34,35]. This is consistent with the results of the present study, in which HS at 42 days of age significantly reduced the body weight of broilers and severely affected the daily feed intake, which led to lower daily weight gain and higher feed-to-weight ratios, while HS caused a significant decrease in the weights of the thymus, spleen, and bursa of Fasciola gigantic, and the organ indices, which suggests that chronic and sustained HS causes severe atrophy of the immune organs of broilers, which severely impairs immune function.

Chronic HS damages the tissue structure of the spleen. Histopathological sections show that HS can blur the boundaries of the spleen’s red and white medulla oblongata, widen the gaps between splenic cells, and loosely arrange the cells. The parenchymal cells of the spleen are necrotic, with the phenomenon of chromatin condensation. Some studies have found that HS can cause cell necrosis in the thymus and spleen, lymphocyte necrosis and vacuolation in the cortical area [36], mild edema in the bursa and spleen, thinning of the cortical area of the thymus, and fewer medullary lymphocytes with nuclear consolidation in chicks after HS [37]. This is consistent with the results of this experimental study, which found that HS can inhibit the normal development of the spleen, causing damage to the spleen and affecting its immune function.

HS can lead to excessive oxidative reactions in the organism, causing oxidative damage to cellular DNA, proteins, and lipids, leading to apoptosis [38,39]. Moreover, oxidative stress is a crucial mechanism for inducing cellular damage in tissues. In the process of oxidative stress, there is an excessive release of MDA, which can cause an increase in cell membrane permeability and reduce ATP activity, leading to cell injury [40]. GSH-PX, SOD, and CAT are important antioxidant factors, and T-AOC can also respond to the antioxidant capacity of the organism. Under a heat-stress environment, the degree of peroxidation of serum increases significantly, accelerating the depletion of serum antioxidant factors and destroying the antioxidant capacity of the organism, which leads to the development of oxidative stress in the organism [41,42]. HS can lead to oxidative stress in many organs and tissues of the body. Several studies have shown that HS causes intestinal oxidative damage by increasing MDA content and decreasing the activities of GSH-Px, CAT, and T-AOC in the mucosa of the duodenum, jejunum, and ileum of rats [43]. HS stimulates an increase in ROS content in bovine mammary epithelial cells, an increase in MDA accumulation, and an inhibition of the expression and activity of the antioxidant enzymes SOD and CAT, leading to mammary inflammation in dairy cows, which negatively affects milk production [44]. This is consistent with the results of the present study that HS severely increased the accumulation of MDA in the serum of the organism and severely reduced the activities of GSH-PX, SOD, and CAT antioxidant factors and decreased T-AOC. HS induced a decrease in the antioxidant capacity of broilers, ultimately leading to oxidative stress and impairing the immune function of the organism.

Immune and inflammatory responses are critical physiological processes regulating the body’s immune function. Abnormal cytokine changes can be used as a primary indicator of the immunosuppressive state [45], which may affect the expression of inflammatory mediators when an extreme stimulus occurs, thus inducing an overall inflammatory response in the body [46]. IL-6, known as a B cell growth factor, plays a crucial role in the growth and development of B cells [47]. IL-4, an essential immunomodulatory factor in the body, induces the maturation of T cells, mast cells, macrophages, and B cells and stimulates the production of immunoglobulins by B cells, which plays a vital role in regulating humoral immunity [48]. TNF-α is also an inflammatory reaction-initiating factor that activates macrophages and monocytes and enhances their killing power [49]. IFN-γ, an immune response-stimulating cytokine, is a key signaling molecule that activates immune cells, which can activate immune responses and inflammation [50]. It has been shown that HS induces overexpression of pro-inflammatory cytokines IL-6, IFN-γ, and TNF-α in broiler intestinal tissues, mediating inflammation [51]. Similar to the results of this experiment, HS was able to induce an increase in the levels of IL-6, IFN-γ, and TNF-α in broiler spleens and increased with duration, whereas IL-4 showed a significant decrease with the onset of HS, which suggests that HS induced an imbalance of cytokine expression in broiler spleens, inducing an immune-inflammatory response in the broiler spleens, which ultimately led to immune dysfunction in the spleen.

The TLR receptor family plays a vital role in natural immunity and inflammation. TLR2 mainly relies on interactions with other TLRs. Studies have shown that combating the activity of TLR2 and TLR4 in rats’ livers or lungs substantially minimizes the severity of local ischemia-reperfusion damage [52]. The expression of TLR4 mRNA in the mononuclear cells of the peripheral blood of heat-stressed pigs is enormously raised. HS can regulate the host immunological responses by modulating the expression of TLR4 [53]. HS causes changes in the production of HSP proteins. HSP70, an essential member within the HSP complex family members, can bind to TLR4 and trigger an inflammation reaction [54]. The signaling pathways of TLR4 are mainly MyD88-dependent and TRIF-dependent signaling pathways [55]. TLR4 triggers the NF-κB signaling pathway through these two transduction routes, initiating an inflammatory response [56]. The experiment’s results illustrated a considerable rise in the transcription of HSP70 mRNA in the spleen of broiler chickens. Additionally, there was a significant increase in mRNA levels for TLR2, TLR4, MYD88, TRIF, and NF-kB genes, which are part of the relevant pathway. These findings indicate that prolonged exposure to high temperatures can trigger an inflammatory reaction in the spleen of broiler chickens by activating the TLRs/MyD88/NF-κB signaling pathway. This, in turn, can result in spleen damage and impaired immune function. The ensuing inflammation in the spleen resulted in damage and impaired immunological function in the broiler spleen.

## 5. Conclusions

In this experiment, chronic Heat Stress (HS) caused a decrease in broiler production performance, a decrease in the body’s antioxidant capacity, and oxidative stress, which led to pathological injuries and a decrease in the immune function of broiler spleens. HS-induced inflammatory reactions in the spleen influenced the production of genes associated with the TLR/MyD88/NF-κB signaling pathway, and these damages were exacerbated as the duration of HS increased. According to the research results, it primarily harms broilers’ spleen by triggering an inflammatory reaction. Therefore, in preventing HS in broilers, in addition to basic methods such as lowering the temperature and ventilation, effective inhibition of the inflammatory response is one of the ways to alleviate HS potentially. The findings of this experiment are crucial for investigating pharmaceuticals that impede the inflammatory reaction to mitigate the harm induced by HS and minimize the losses incurred in broiler breeding due to HS.

## Figures and Tables

**Table 1:** Composition of the basal diet.

Nutritional Indicators (%)	Day of Age	
	0–21 Days of Age	21–42 Days of Age
Padding≤	14	14
Crude protein≥	21.5	21
Crude fiber≤	2	5
Crude ash≤	7	8
Calcium	0.7–1.4	0.7–1.4
Total phosphorus≥	0.5	0.5
Salt	0.3–0.8	0.3–0.8
Methionine	0.3–0.8	0.3–0.8

**Table 2:** Information of sequences of the oligonucleotide primers.

Gene	Sequences (5′-3′)
β-actin F	CCGCTCTATGAAGGCTACGC
β-actin R	CTCTCGGCTGTGGTGGTGAA
TLR2 F	GGTGGCCAGAAAGCTACATC
TLR2 R	GGGTGCAGATCAAGGACACT
TLR4 F	CCAAACACCACCCTGGACTT
TLR4 R	CCATGGAAGGCTGCTAGACC
MYD88 F	GAGGGATGATCCGTATGGGC
MYD88 R	ACACGTTCCTGGCAAGACAT
NF-κB F	ACACCACTGGATATGGCAGC
NF-κB R	TCTTGCTTGGATCAGGCGTT
TRIF F	CCCAGTGTCTGTCTCTGCTG
TRIF R	GTTGTGTAGTGCTGGCCTGA
IL-4 F	TGCTTACAGCTCTCAGTGCC
IL-4 R	TCTTGACGCAGGAAACCTCTC
IL-6 F	CTCGTCCGGAACAACCTCAA
IL-6 R	TCAGGCATTTCTCCTCGTCG
TNF-α F	CAGATGGGAAGGGAATGAAC
TNF-α R	AGAGCATCAACGCAAAAGGG
IFN-γ F	GCTGACGGTGGACCTATTATTGTAGAG
IFN-γ R	TTCTTCACGCCATCAGGAAGGTTG
HSP70-1 F	TGTGGCCTTCACCGATACAG
HSP70-1 R	TGGGGTCATCATACTTGCGG

**Table 3:** Effect of chronic HS on broiler performance.

Production Performance	Group		*p*-Value
	HS	Control	
Weight at 21 days of age (g)	942.40 ± 37.26	939.60 ± 43.59	0.916
Weight at 42 days of age (g)	2377.60 ± 37.27	2682.80 ± 27.35	<0.01
ADG (g)	68.82 ± 1.75	83.01 ± 1.45	<0.01
ADFI (g)	122.50 ± 2.69	138.10 ± 2.45	<0.01
F/G	1.79 ± 0.49	1.66 ± 0.30	<0.01

Data are presented as mean ± SE (n = 5), HS: Heat stress group, Control: control group.

**Table 4:** Effect of chronic HS on immune organ index in broilers.

Items	Group		*p*-Value
	HS	Control	
Thymus index	2.465 ± 0.11	2.563 ± 0.68	0.014
Spleen index	1.237 ± 0.09	1.286 ± 0.31	<0.01
Fasciola index	0.648± 0.011	0.708 ± 0.016	<0.01

Data are presented as mean ± SE (n = 5), HS: Heat stress group, Control: control group.

## Data Availability

The data presented in this study are available on request from the corresponding author.
